# Comparison of Invasive Pancreatic Ductal Adenocarcinoma versus Intraductal Papillary Mucinous Neoplasm: A National Cancer Database Analysis †

**DOI:** 10.3390/cancers15041185

**Published:** 2023-02-13

**Authors:** Ioannis A. Ziogas, Salvador Rodriguez Franco, Nicholas Schmoke, Cheryl Meguid, Cassandra Murphy, Mohammed Al-Musawi, Sophoclis P. Alexopoulos, Richard D. Schulick, Marco Del Chiaro

**Affiliations:** 1 Division of Surgical Oncology, Department of Surgery, University of Colorado, Anschutz Medical Campus, Aurora, CO 80045, USA; 2 Division of Hepatobiliary Surgery and Liver Transplantation, Department of Surgery, Vanderbilt University Medical Center, Nashville, TN 37232, USA; 3 The Heart Institute, Children’s Hospital Colorado, Aurora, CO 80045, USA; 4 Clinical Trials Office, Department of Surgery, University of Colorado, Anschutz Medical Campus, Aurora, CO 80045, USA; 5 University of Colorado Cancer Center, Anschutz Medical Campus, Aurora, CO 80045, USA

**Keywords:** pancreatic ductal adenocarcinoma, intraductal papillary mucinous neoplasm, invasive carcinoma, National Cancer Database, survival

## Abstract

**Simple Summary:**

This study aimed to compare the characteristics, management, and overall survival of pancreatic ductal adenocarcinoma (PDAC) vs. invasive intraductal papillary mucinous neoplasm (IPMN) using the National Cancer Database in the US. This study included 101,190 patients, with 100,834 having PDAC and 356 having IPMN. The results showed that PDAC was more aggressive than IPMN, with a lower proportion of patients undergoing surgery but a higher proportion receiving chemotherapy or radiation. The median overall survival for PDAC was 8.3 months and for IPMN it was 33.4 months. Surgery was found to improve overall survival, and efforts should focus on facilitating surgical treatment for better outcomes.

**Abstract:**

Background: Current evidence on overall survival (OS) between invasive pancreatic ductal adenocarcinoma (PDAC) and intraductal papillary mucinous neoplasm (IPMN) is limited to single-center reports. We aimed to compare the characteristics, management, and OS of invasive PDAC vs. IPMN using a national United States (US) database. Methods: Invasive PDAC or IPMN adult (≥18 years) patients were identified in the National Cancer Database (2004–2016). OS was assessed with the Kaplan–Meier method and the stratified log-rank test. Results: We included 101,190 patients (100,834 PDAC, 356 IPMN). A higher proportion of PDAC vs. IPMN patients had clinical N1 (36.8% vs. 15.7%, *p* < 0.001) and M1 disease (41.2% vs. 5.9%, *p* < 0.001). A lower proportion of PDAC patients underwent surgery (25.5% vs. 80.3%, *p* < 0.001), but a higher proportion received chemotherapy (65.4% vs. 46.1%, *p* < 0.001) or radiation (25.3% vs. 20.5%, *p* = 0.04). A higher proportion of surgical patients with PDAC vs. IPMN underwent margin-positive resection (23.0% vs. 14.0%, *p* = 0.001). The median OS for PDAC vs. IPMN was 8.3 vs. 33.4 months. In the stratified analysis for N0M0 disease, the median OS for PDAC vs. IPMN was 12.8 vs. 43.3 months, for N1M0, it was 11.5 vs. 17.0 months, while for M1, it was 4.0 vs. 7.0 months. In both diagnoses, surgery yielded improved OS, while stratified analysis in the surgical cohort demonstrated similar findings. Conclusions: Invasive PDAC is more aggressive than invasive IPMN, yet in the case of metastasis, OS is equally poor. Excellent long-term OS is achievable with surgical resection in highly selected cases, and efforts should focus on facilitating surgical treatment.

## 1. Introduction

Pancreatic ductal adenocarcinoma (PDAC) is the most common type of pancreatic cancer, and by 2030, it is expected to constitute the second most common cause of cancer-related mortality in the United States [1]. Intraductal papillary mucinous neoplasm (IPMN) is a benign precancerous lesion with different disease biology and a broad spectrum of presentation that ranges from benign adenoma to invasive carcinoma. The prevalence of IPMN in the general population ranges between 3 and 10% in older adults, according to different studies [2,3,4]. However, more recent and dedicated prospective studies have shown a prevalence of pancreatic cystic lesions of around 50%, suggesting that IPMN prevalence can be higher than previously reported [5]. Given the wider availability of high-quality imaging, an increasing number of individuals are diagnosed with IPMN [6]. Due to IPMN’s potential for malignant transformation, either surveillance or surgical resection is employed [7].

As part of its natural history, IPMN can progress to high-grade dysplasia and then to invasive carcinoma, also known as IPMN with an associated invasive carcinoma. Malignant IPMNs are reported in about 11–33% of resected branch-duct IPMNs and in about 36–100% of main-duct/mixed-type IPMNs [8]. In comparison with PDAC, invasive IPMN harbors certain clinicopathological characteristics and debatable outcomes. In fact, the currently available comparative studies on PDAC vs. IPMN are limited to single- or bi-center reports or span over an older era [9,10,11,12]. The selection between surgical resection and surveillance for IPMN management remains a matter of debate. For high-grade dysplasia or invasive main-duct-type IPMN, surgical resection is widely accepted as the standard of care, whereas for branch-duct and mixed-type IPMNs, the appropriate approach is less clear. Although dilation ≥5 mm has been linked with malignancy, the commonly used cutoff for surgical resection remains at ≥10 mm, further emphasizing the lack of standardization in IPMN treatment [7,8].

In this study, we aimed to compare the characteristics, management, and overall survival (OS) of patients with invasive PDAC vs. patients with invasive IPMN using national US data over a contemporary era.

## 2. Materials and Methods

### 2.1. Data Source and Patient Population

All patients with invasive PDAC or invasive IPMN who were recorded in the National Cancer Database (NCDB) between 2004 and 2016 were included in this study. Histopathologic confirmation was not mandatory for patient inclusion in this study. About 70% of all newly diagnosed malignancies at more than 1500 hospitals in the United States are included in the NCDB [13]. Data on demographics, clinicopathological characteristics, tumor features, treatment, and survival are also included in this database [14].

**Figure 1:**
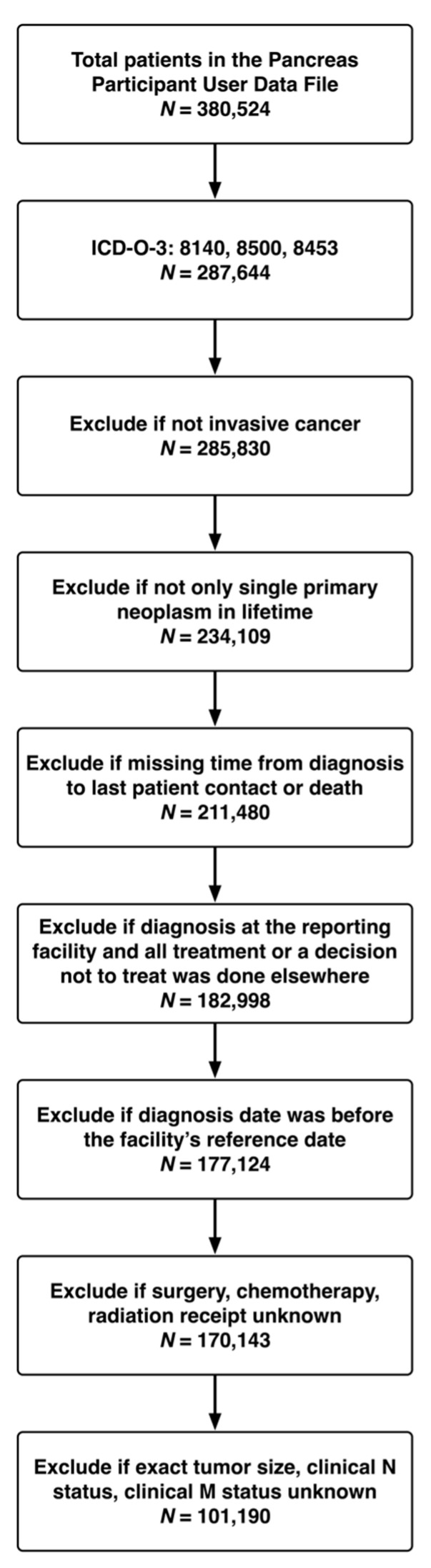
Assembly of analysis cohort.

In the current study, we identified adults (≥18 years) with PDAC or invasive IPMN using the International Classification of Diseases for Oncology, 3rd Edition, histology codes “8140” and “8500” for PDAC and “8453” for IPMN. Figure 1 depicts our cohort assembly. The University of Colorado Institutional Review Board stated that no official waiver was needed since no patient, physician, or hospital identifiers were examined as well as the data were de-identified.

### 2.2. Covariates and Outcome

We extracted the following patient demographic data: age, sex, and race. Clinicopathological and treatment-related data extracted included primary site of pancreatic lesion, tumor size, clinical nodal (N) status, clinical metastatic (M) status, receipt of surgery, type of surgery (i—local excision/pancreatectomy/surgery, not otherwise specified; ii—partial pancreatectomy (i.e., distal); iii—local/partial pancreaticoduodenectomy (PD); iv—total pancreatectomy/extended PD), resection margin status (margin-negative resection (R-), margin-positive resection (R+), or unknown margin status), receipt of chemotherapy, receipt of radiation, and reason for no surgery. The primary outcome of interest, OS, was calculated as the amount of time in months between the date of diagnosis and the date of last patient contact or death.

### 2.3. Statistical Analysis

Continuous data were summarized as medians and interquartile ranges (IQRs), while categorical data were reported as frequencies and percentages. Univariable analyses were conducted using the Mann–Whiney U test or the Chi-square test, depending on the variable’s nature. Kaplan–Meier and stratified log-rank analyses were implemented for survival analysis. Hazard ratio and 95% confidence intervals (CIs) were also calculated using univariable and multivariable Cox regression models. All statistical analyses were performed using Stata IC 16.0 (StataCorp LLC, College Station, Texas). Significance was set at a *p*-value of less than 0.05 for all two-sided statistical tests.

## 3. Results

### 3.1. Patient Characteristics

A total of 101,190 patients—100,834 with invasive PDAC and 356 with invasive IPMN—were included. No statistically significant difference was observed between the two groups regarding age, sex, and race. A higher proportion of invasive PDACs were present in the body of the pancreas compared with invasive IPMNs (*p <* 0.001). Patients with PDAC had slightly larger tumors than patients with IPMN (*p =* 0.004). Compared to IPMN patients, a significantly higher percentage of PDAC patients had clinical N1 status (36.8% vs. 15.7%, *p <* 0.001), M1 disease (41.2% vs. 5.9%, *p <* 0.001), and received chemotherapy (65.4% vs. 46.1%, *p <* 0.001) or radiation (25.3% vs. 20.5%, *p =* 0.04). A lower proportion of PDAC patients underwent surgery (25.5% vs. 80.3%, *p <* 0.001). Detailed characteristics of the entire cohort are presented in Table 1.

A total of 25,702 patients with invasive PDAC and 286 with invasive IPMN underwent surgical treatment. Age, sex, race, and tumor size did not differ between the groups. Tumor location in the tail of the pancreas was less frequent for PDACs (*p <* 0.001). PDAC patients had, more often, clinical N1 (28.9% vs. 12.6%, *p <* 0.001) and M1 disease (3.3% vs. 1.4%, *p <* 0.001) compared with IPMN patients. Similar to the entire cohort, a higher proportion of PDAC patients received chemotherapy (75.8% vs. 48.3%, *p <* 0.001) or radiation (40.0% vs. 19.2%, *p =* 0.04) compared to IPMN patients. A lower proportion of PDAC patients underwent distal pancreatectomy or total pancreatectomy/extended PD, while a higher proportion underwent a local/partial PD (*p =* 0.002) compared with IPMN patients. A higher proportion of PDAC patients underwent R+ (23.0% vs. 14.0%, *p =* 0.001) compared with IPMN patients. Detailed characteristics of the surgical cohort are presented in Table 2.

### 3.2. Overall Survival

**Figure 2:**
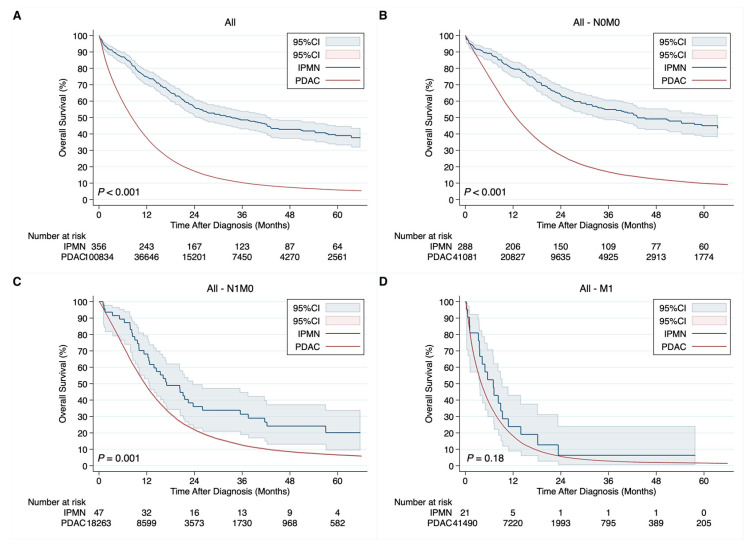
Kaplan–Meier curves demonstrating differences in overall survival in the entire cohort for invasive pancreatic ductal adenocarcinoma (PDAC) vs. invasive intraductal papillary mucinous neoplasm (IPMN) overall (**A**), in the subgroup of N0M0 disease (**B**), in the subgroup of N1M0 disease (**C**), and in the subgroup of M1 disease (**D**).

In the entire cohort, the median OS for PDAC was 8.3 vs. 33.4 months for IPMN (Figure 2A). In univariable Cox regression, patients with invasive PDAC had 3.01 times higher overall mortality risk than patients with invasive IPMN (95%CI: 2.63–3.46, *p <* 0.001). In stratified analysis, for N0M0 the median OS for PDAC vs. IPMN was 12.8 vs. 43.3 months (Figure 2B), for N1M0, it was 11.5 vs. 17.0 months (Figure 2C), and for M1, it was 4.0 vs. 7.0 months (Figure 2D). In both, patients with PDAC and patients with IPMN, undergoing surgical treatment was associated with a survival benefit (both *p <* 0.001). In multivariable Cox regression adjusted for age, sex, race, tumor size, the primary site of pancreatic lesion, clinical N status, clinical M status, the receipt of surgery, chemotherapy, and radiation, patients with invasive PDAC had 2.48 times higher risk of overall mortality compared with patients with invasive IPMN (95%CI: 2.16–2.84, *p <* 0.001).

**Figure 3:**
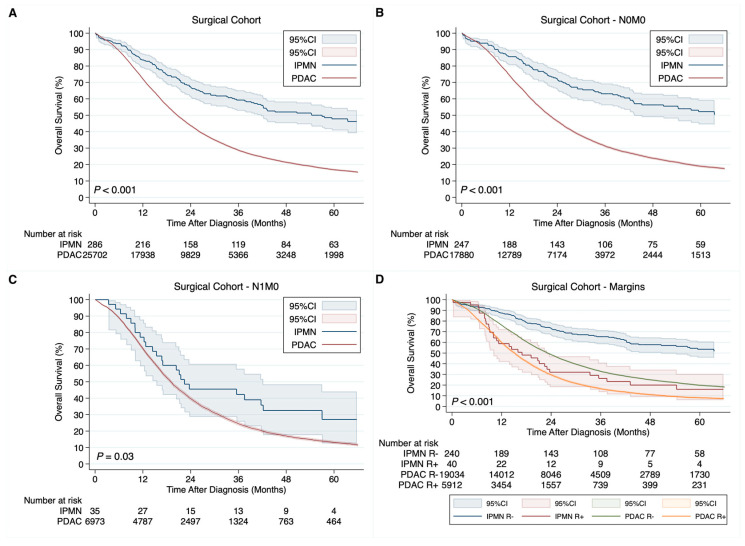
Kaplan–Meier curves demonstrating differences in overall survival in the surgical cohort for invasive pancreatic ductal adenocarcinoma (PDAC) vs. invasive intraductal papillary mucinous neoplasm (IPMN) overall (**A**), in the subgroup of N0M0 disease (**B**), in the subgroup of N1M0 disease (**C**), and by resection margin status (**D**).

In the surgical cohort, the median OS for PDAC vs. IPMN was 20.6 vs. 57.0 months (Figure 3A). In univariable Cox regression, patients with invasive PDAC had 2.25 times higher risk of overall mortality compared with patients with invasive IPMN (95%CI: 1.90–2.65, *p <* 0.001). In stratified analysis, for N0M0 disease, the median OS for PDAC vs. IPMN was 22.0 vs. 84.9 months (Figure 3B), and for N1M0 disease, it was 19.0 vs. 22.4 months (Figure 3C). The median OS for PDAC patients undergoing R- and R+ was 23.2 and 15.2 months, while for IPMN patients undergoing R- and R+, it was 85.1 and 17.0 months (Figure 3D). In multivariable Cox regression adjusted for age, sex, race, tumor size, the primary site of pancreatic lesion, clinical N status, clinical M status, the type of surgery, resection margin status, chemotherapy, and radiation, patients with invasive PDAC had 2.55 times higher risk of overall mortality compared with patients with invasive IPMN (95%CI: 2.16–3.02, *p <* 0.001).

## 4. Discussion

The present study showed that patients with invasive PDAC demonstrate inferior OS compared with patients with invasive IPMN in the absence of metastasis, while in the case of metastasis, the outcomes are equally poor for either diagnosis. Since surgery, and particularly R- resection, is associated with improved OS, improving the access of patients to experienced pancreas centers that can offer surgical treatment options is of paramount importance. Nonetheless, even among surgically treated patients, those with non-metastatic invasive IPMN tend to survive longer than those with non-metastatic invasive PDAC.

Our findings of superior survival for surgically treated patients with invasive IPMN compared with PDAC, even in more advanced disease stages, align with a multicenter study from Japan [9]. However, their median survival of 46 months for IPMN and 12 months for PDAC are shorter than the 57 months for IPMN and 21 months for PDAC in our study. In a bi-center report from 2011, Waters et al. [10] reported their 10-year experience of resecting invasive PDAC vs. IPMN from Mayo Clinic and Indiana University Hospital. Similar to our findings, the authors showed that invasive IPMN patients had better OS compared with PDAC patients overall and in N0 disease [10]. Although they did not observe a significant difference in OS between IPMN and PDAC in the case of N1 disease [10], our findings are consistent with the presence of a potential survival benefit for IPMN even in that disease stage. A matched control study from Memorial Sloan Kettering Cancer Center highlighted the better OS for colloid invasive IPMN compared with either tubular invasive IPMN or conventional PDAC, while the latter two entities had similar OS [15]. Notably, the authors found no difference in OS between colloid invasive IPMN and PDAC in the case of N1 disease, while they found a survival benefit for tubular invasive IPMN over PDAC in the case of N0 disease, hence, underlining the prognostic importance of nodal disease status [15].

In a retrospective comparison of invasive IPMN vs. PDAC from Nagoya University, Japan, the authors showed improved OS for early-stage invasive IPMN vs. PDAC but no difference in OS for advanced-stage disease, while disease-free survival did not differ in either disease stage [16]. Similarly, a 2019 systematic review and meta-analysis demonstrated the superior 5-year OS of invasive IPMN compared with PDAC overall, which was mitigated in the setting of advanced-stage disease [17]. Additionally, a recent study from Karolinska University Hospital, Sweden, showed superior unadjusted OS for invasive IPMN, while adjusting for covariates yielded no difference in OS between invasive IPMN and PDAC [11]. One potential explanation for the difference in our findings compared with the study from Karolinska is the higher proportion of patients with advanced-stage disease in their study compared with our study. For instance, 82% of IPMNs and 92% of PDACs in their study had positive nodal disease compared with only 13% and 29% in our study, while 17% of both IPMNs and PDACs in their study had metastasis compared with only 1% of IPMNs and 3% of PDACs in our study. Given the growing body of evidence showing more similar outcomes between invasive IPMN and PDAC in advanced-stage disease, the differences listed above regarding these poor prognostic indicators may account for the discrepancy between our findings and those from Karolinska [11].

Our analysis has certain limitations mostly related to retrospective database analysis, such as the potential for selection bias. The lack of certain variables of interest from NCDB (e.g., IPMN subtype, main-duct vs. branch-duct IPMN, perineural invasion, and recurrence data) or the high percentage of missing data (e.g., grade/differentiation, 60.5% missing/unknown data; lymph-vascular invasion, 78.8% missing/unknown data; and carbohydrate antigen 19-9 level, 83.3% missing/unknown exact level) did not allow us to analyze that data. A potential explanation for the improved OS in invasive IPMN patients could be the more intensive surveillance protocols employed for these patients, as well as the potential for lead-time bias compared with invasive PDAC patients.

## 5. Conclusions

In conclusion, the findings of this large US database analysis suggest a significantly better OS for invasive IPMN compared with invasive PDAC both in the entire cohort and in surgically treated patients, while in the case of distant metastasis, the outcomes are equally poor. This highlights the potentially more aggressive disease biology of invasive PDAC compared to invasive IPMN, yet the retrospective nature of data and lack of in-depth data granularity limits our ability to deduce robust conclusions. Prospective comparative studies might provide further insight in the future. Nevertheless, excellent long-term OS was observed with surgical resection in highly selected cases; attention should be directed toward streamlining surgical treatment.

## Figures and Tables

**Table 1:** Demographics by tumor type in all patients.

Variable	PDAC (*n* = 100,834)	IPMN (*n* = 356)	*p*-Value
Age (years)	67.0 (59.0–76.0)	69.0 (60.0–76.0)	0.29
Sex			0.48
Male	51,063 (50.6%)	187 (52.5%)	
Female	49,771 (49.4%)	169 (47.5%)	
Race			0.10
White	83,411 (82.7%)	305 (85.7%)	
Black	12,775 (12.7%)	32 (9.0%)	
Other/unknown	4648 (4.6%)	19 (5.3%)	
Primary site of the pancreatic lesion			<0.001
Head	58,211 (57.7%)	210 (59.0%)	
Body	13,767 (13.7%)	26 (7.3%)	
Tail	12,570 (12.5%)	39 (11.0%)	
Other	16,286 (16.2%)	81 (22.8%)	
Tumor size (cm)	3.6 (2.8–4.7)	3.5 (2.0–5.3)	0.004
Clinical N status			<0.001
N0	63,689 (63.2%)	300 (84.3%)	
N1	37,145 (36.8%)	56 (15.7%)	
Clinical M status			<0.001
M0	59,344 (58.9%)	335 (94.1%)	
M1	41,490 (41.2%)	21 (5.9%)	
Surgery			<0.001
No	75,132 (74.5%)	70 (19.7%)	
Yes	25,702 (25.5%)	286 (80.3%)	
Chemotherapy			<0.001
No	34,921 (34.6%)	192 (53.9%)	
Yes	65,913 (65.4%)	164 (46.1%)	
Radiation therapy			0.04
No	75,328 (74.7%)	283 (79.5%)	
Yes	25,506 (25.3%)	73 (20.5%)	
Reason for no surgery			<0.001
Surgery was performed	25,702 (25.5%)	286 (80.3%)	
It was not part of the planned treatment	63,835 (63.3%)	49 (13.8%)	
Contraindicated due to patient risk factors	8016 (8.0%)	14 (3.9%)	
Patient died prior to surgery	349 (0.4%)	1 (0.3%)	
Surgery was refused	1269 (1.3%)	4 (1.1%)	
No reason described/recorded	359 (0.4%)	2 (0.6%)	
Unknown	1304 (1.3%)	0 (0.0%)	

**Table 2:** Demographics by tumor type in the surgical cohort.

Variable	PDAC (*n* = 25,702)	IPMN (*n* = 286)	*p*-Value
Age (years)	66.0 (58.0–73.0)	67.0 (59.0–74.0)	0.07
Sex			0.12
Male	13,021 (50.7%)	158 (55.2%)	
Female	12,681 (49.3%)	128 (44.8%)	
Race			0.29
White	21,991 (85.6%)	245 (85.7%)	
Black	2593 (10.1%)	24 (8.4%)	
Other/Unknown	1118 (4.4%)	17 (5.9%)	
Primary site of the pancreatic lesion			<0.001
Head	18,907 (73.6%)	167 (58.4%)	
Body	1835 (7.1%)	19 (6.6%)	
Tail	2391 (9.3%)	38 (13.3%)	
Other	2569 (10.0%)	62 (21.7%)	
Tumor size (cm)	3.1 (2.5–4.0)	3.4 (1.9–5.0)	0.89
Clinical N status			<0.001
N0	18,281 (71.1%)	250 (87.4%)	
N1	7421 (28.9%)	36 (12.6%)	
Clinical M status			<0.001
M0	24,853 (96.7%)	282 (98.6%)	
M1	849 (3.3%)	4 (1.4%)	
Chemotherapy			<0.001
No	6228 (24.2%)	148 (51.8%)	
Yes	19,474 (75.8%)	138 (48.3%)	
Radiation therapy			<0.001
No	15,427 (60.0%)	231 (80.8%)	
Yes	10,275 (40.0%)	55 (19.2%)	
Type of surgery			0.002
Local excision/pancreatectomy/surgery, not otherwise specified	993 (3.9%)	8 (2.8%)	
Partial pancreatectomy (i.e., distal)	3388 (13.2%)	50 (17.5%)	
Local/partial PD	16,591 (64.6%)	157 (54.9%)	
Total pancreatectomy/extended PD	4730 (18.4%)	71 (24.8%)	
Surgical margin status			0.001
R-	19,034 (74.1%)	240 (83.9%)	
R+	5912 (23.0%)	40 (14.0%)	
No evaluable/unknown	756 (2.9%)	6 (2.1%)	

PD: pancreaticoduodenectomy; R-: margin-negative resection; and R+: margin-positive resection.

## Data Availability

The data that support the findings of this study are available from the Commission on Cancer’s National Cancer Database (NCDB). They are de-identified patient-level data that do not identify hospitals, health care providers, or patients as agreed to in the Business Associate Agreement that each Commission on Cancer accredited program has signed with the American College of Surgeons. Restrictions apply to the availability of these data, which were used under license for this study. Data are available at https://www.facs.org/quality-programs/cancer/ncdb/puf with the permission of the American College of Surgeons (accessed on 1 September 2021).
